# Know Thy Kit—Why Catheter Design Matters!

**DOI:** 10.5152/tjg.2025.0201255

**Published:** 2025-08-01

**Authors:** Henriette Heinrich

**Affiliations:** Department of Gastroenterology and Hepatology, University Digestive Health Care Center Basel-Clarunis, Basel, Switzerland

High-resolution esophageal manometry (HRM) has transformed our understanding of esophageal motility by turning isolated point measurements into seamless pressure topographies that span from the upper esophageal sphincter through the lower esophageal sphincter to the stomach. In its earliest iterations, conventional manometry relied on few water-perfused (WP) channels prone to movement artifacts and offered limited spatial resolution. The advent of densely arrayed solid-state (SS) sensors ushered in the era of Clouse plots and served as the foundation for the Chicago Classification, now in its fourth iteration.^1,2^ Despite these advances, WP HRM catheters remain in widespread clinical use, particularly in environments where cost constraints or concerns about catheter fragility and hygiene limit the deployment of SS technology.^3^ However, comparative data between SS and WP systems within the same individuals were lacking.

The current paper by Bor et al entitled “Normal Values in Esophageal High-Resolution Manometry Performed Using 36-Channel Water-Perfused Catheter or Solid-State Catheter” addresses this gap.^4^ Healthy volunteers underwent both SS and WP studies on back-to-back days in a randomized order. Applying an identical supine protocol of ten 5 mL water swallows, the researchers measured resting pressures at the lower and upper esophageal sphincters, calculated the 4-second integrated relaxation pressure (IRP), assessed the distal contractile integral (DCI), recorded distal latency, and quantified the proportion of ineffective swallows. Because each subject served as their own control, inter-individual variability was minimized, allowing clear attribution of observed differences to the catheter systems themselves rather than to underlying physiological heterogeneity.

Analysis of the resulting data revealed that SS catheters tended to register higher contractile vigor and more pronounced relaxation nadirs, whereas WP systems demonstrated attenuated peaks and shallower nadirs. Median IRP values derived from SS technology were markedly higher than those from WP recordings, and DCI ranges were also substantially increased when measured with SS sensors. Resting sphincter pressures followed the same pattern, with WP readings averaging roughly half of those obtained via SS catheters. These findings underscore the risk of misclassification if SS-based thresholds are indiscriminately applied to WP studies, potentially leading to overdiagnosis of outflow obstruction or underrecognition of hypocontractility.

This study builds upon and refines earlier normative WP SS datasets. In 1 European cohort, WP IRP values and DCI ranges were reported to overlap substantially with SS norms, suggesting minimal bias under certain conditions. In contrast, research in an Asian population found WP metrics that more closely mirrored the current study’s IRP results but diverged at the extremes of DCI, illustrating how technical factors shape normative thresholds. A systematic review encompassing over 50 HRM studies further documented wide variability attributable to catheter type, sensor spacing, patient posture, bolus characteristics, and subject demographics.^5-10^ The within-subject design employed in the Turkish work offers a rare lens through which to isolate technical contributions from true physiologic variance.

Key aspects of sensor design and spacing help explain these systematic differences. Solid-state technology, utilizing circumferential strain gauges or piezoelectric elements, captures rapid pressure changes with minimal damping and averages signals around the catheter circumference, which produces sharp contractile peaks and deep relaxation nadirs. In WP systems, by contrast, pressure transmission occurs through a column of perfused fluid whose inertia smooths out transient events, leading to lower peak DCIs and less distinct IRP nadirs, and sensors are unidirectionally located. Further, high-density sensor spacing ensures that brief peristaltic breaks and the full extent of a contraction wave contribute to DCI calculations, whereas wider spacing between sensors can under-sample localized gaps and misplace maximal contraction points.

Individual patient characteristics further affect HRM measurements. Age-related changes in smooth muscle function and neural control tend to reduce contractile vigor and increase the prevalence of ineffective swallows. Sex-related differences often manifest as modest variations in DCI and IRP, potentially linked to hormonal influences and differences in muscle mass. Body mass index can affect intra-abdominal pressure and resting lower esophageal sphincter tone. Coexisting conditions such as diabetes or connective tissue diseases may disrupt neuromuscular coordination, prolonging distension latency, and diminishing contractile force. Even under standardized conditions, factors including bolus temperature, swallow coaching, and patient comfort contribute to observed variability. Accordingly, normative datasets achieve their greatest clinical relevance when stratified by age, sex, and body mass and when strict adherence to procedural protocols is maintained.^11,12^

Although this study represents a meaningful advance in catheter-specific normative HRM, certain elements suggest areas for further exploration. The volunteer cohort, comprising just over 30 subjects undergoing both catheter types and 40 enrolled for WP measurements, came from a single institution and featured a male-predominant sample in the fourth decade of life. Expanding such comparisons to include pediatric, elderly, and female-predominant populations would strengthen the precision and applicability of these reference ranges. While the supine water swallow protocol aligns with many clinical practices, the incorporation of upright and solid bolus assessments could more comprehensively reflect everyday eating conditions which are reflected in the new version of the Chicago Classification.

Looking forward, harmonizing HRM interpretation across modalities calls for collaborative efforts to develop and disseminate catheter-specific normative tables, to mandate detailed documentation of parameters in HRM reporting, and to design normative studies that embrace a wide spectrum of patient ages, sexes, and body compositions. Extending protocols to include positional and bolus variability will enhance cross-center comparability. Crucially, prospective studies validating these advanced thresholds against clinical outcomes will ensure that nominal “normal” ranges translate into better diagnostic accuracy and more tailored therapeutic strategies. By integrating the technical insights of sensor design with an appreciation for patient physiology, the field can move closer to universally robust HRM standards that improve care for those with esophageal motility disorders.

Most importantly, this comprehensive study highlights that practitioners of esophageal HRM need to know their kit and use specific normal values to ensure correct diagnostic classification of patients.

## Data Availability

The data that support the findings of this study are available on request from the corresponding author.
